# Conditioned medium from adipose-derived stem cells attenuates ischemia/reperfusion-induced cardiac injury through the microRNA-221/222/PUMA/ETS-1 pathway

**DOI:** 10.7150/thno.52677

**Published:** 2021-01-01

**Authors:** Tzu-Lin Lee, Tsai-Chun Lai, Shu-Rung Lin, Shu-Wha Lin, Yu-Chen Chen, Chi-Ming Pu, I-Ta Lee, Jaw-Shiun Tsai, Chiang-Wen Lee, Yuh-Lien Chen

**Affiliations:** 1 Department of Anatomy and Cell Biology, College of Medicine, National Taiwan University, Taipei, Taiwan.; 2 Department of Bioscience Technology, College of Science, Chung-Yuan Christian University, Taoyuan, Taiwan.; 3 Center for Nanotechnology and Center for Biomedical Technology, Chung-Yuan Christian University, Taoyuan, Taiwan.; 4 Department of Clinical Laboratory Sciences and Medical Biotechnology, College of Medicine, National Taiwan University, Taipei, Taiwan.; 5 Department of Anatomy and Cell Biology, College of Medicine, National Taiwan University, Taipei, Taiwan.; 6 Division of Plastic Surgery, Department of Surgery, Cathay General Hospital, Taipei, Taiwan.; 7 School of Dentistry, College of Oral Medicine, Taipei Medical University, Taipei, Taiwan.; 8 Department of Family Medicine, National Taiwan University Hospital, Taipei, Taiwan.; 9 Center for Complementary and Integrated Medicine, National Taiwan University Hospital, Taipei, Taiwan.; 10 Department of Nursing, Division of Basic Medical Sciences, and Chronic Diseases and Health Promotion Research Center, Chang Gung University of Science and Technology, Chiayi, Taiwan.; 11 Research Center for Industry of Human Ecology and Research Center for Chinese Herbal Medicine, Chang Gung University of Science and Technology, Taoyuan, Taiwan.; 12 Department of Orthopaedic Surgery, Chang Gung Memorial Hospital, Chiayi, Taiwan.

**Keywords:** Ischemia/reperfusion injury, ADSC-CM, miR-221/222, apoptosis, fibrosis

## Abstract

**Rationale:** Cardiovascular diseases, such as myocardial infarction (MI), are the leading causes of death worldwide. Reperfusion therapy is the common standard treatment for MI. However, myocardial ischemia/reperfusion (I/R) causes cardiomyocyte injury, including apoptosis and fibrosis. We aimed to investigate the effects of conditioned medium from adipose-derived stem cells (ADSC-CM) on apoptosis and fibrosis in I/R-treated hearts and hypoxia/reoxygenation (H/R)-treated cardiomyocytes and the underlying mechanisms.

**Methods:** ADSC-CM was collected from ADSCs. The effects of intramuscular injection of ADSC-CM on cardiac function, cardiac apoptosis, and fibrosis examined by echocardiography, Evans blue/TTC staining, TUNEL assay, and Masson's trichrome staining in I/R-treated mice. We also examined the effects of ADSC-CM on apoptosis and fibrosis in H/R-treated H9c2 cells by annexin V/PI flow cytometry, TUNEL assay, and immunocytochemistry.

**Results:** ADSC-CM treatment significantly reduced heart damage and fibrosis of I/R-treated mice and H/R-treated cardiomyocytes. In addition, the expression of apoptosis-related proteins, such as p53 upregulated modulator of apoptosis (PUMA), p-p53 and B-cell lymphoma 2 (BCL2), as well as the fibrosis-related proteins ETS-1, fibronectin and collagen 3, were significantly reduced by ADSC-CM treatment. Moreover, we demonstrated that ADSC-CM contains a large amount of miR-221/222, which can target and regulate PUMA or ETS-1 protein levels. Furthermore, the knockdown of PUMA and ETS-1 decreased the induction of apoptosis and fibrosis, respectively. MiR-221/222 overexpression achieved similar results. We also observed that cardiac I/R markedly increased apoptosis and fibrosis in miR-221/222 knockout (KO) mice, while ADSC-CM decreased these effects. The increased phosphorylation of p38 and NF‐κB not only mediated myocardial apoptosis through the PUMA/p53/BCL2 pathway but also regulated fibrosis through the ETS-1/fibronectin/collagen 3 pathway.

**Conclusions:** Overall, our results show that ADSC-CM attenuates cardiac apoptosis and fibrosis by reducing PUMA and ETS-1 expression, respectively. The protective effect is mediated via the miR-221/222/p38/NF-κB pathway.

## Introduction

Cardiovascular diseases are major health problems and associated with clinical mortality worldwide. The most common cardiovascular disease is myocardial ischemia caused by coronary artery occlusion. An effective treatment of myocardial infarction is reperfusion therapy, which can promote blood restoration to the ischemic myocardium. However, this treatment can cause ischemia/reperfusion (I/R) injury, which leads to secondary and complicated heart damage 1. Studies have shown that cardiac apoptosis and fibrosis play important roles in the progression of myocardial I/R injury 2, 3. Cardiomyocyte apoptosis is a rare event in the healthy myocardium. However, this is the earliest and main form of infarcted cardiomyocyte death and is associated with I/R injury 3. PUMA (p53-upregulated modulator of apoptosis) is the only BH3 protein in the BCL2 family that can rapidly induce apoptosis by increasing p53 levels 2, 4. In addition, myocardial fibrosis is caused by excessive extracellular matrix deposition (such as collagen) and activation of myofibroblasts in the damaged area, resulting in scar formation and permanent damage to heart function 5. ETS-1 is an important mediator of cardiac fibrosis induced by angiotensin II 6. However, the mechanism of I/R-induced injury is unclear, and a satisfactory treatment method is not yet available. Preventing cardiac apoptosis and fibrosis has become the goal of certain therapies that interfere with I/R injury.

The use of mesenchymal stem cells (MSCs) for the treatment of I/R injury seems to have many advantages because these cells are safe, pluripotent and have immunological privilege 7, 8. Adipose-derived stem cells (ADSCs) have become the preferred cell type for the treatment of I/R injury because they have the following advantages over other types of MSCs: abundance and expansion ability, relatively easy harvest, high MSC frequency, multi-lineage differentiation, and powerful proliferation 7. There is increasing evidence that ADSC transplantation can improve heart function after myocardial infarction through direct differentiation and paracrine effects 9. The beneficial effect induced by ADSCs is mainly mediated by a paracrine mechanism, and the direct differentiation of cells plays a minor role 10. Paracrine products of ADSCs, including cytokines, growth factors, RNA and microRNAs, may affect cell functions and be delivered to target organs for repair 11. However, the evidence supporting paracrine mechanisms playing a therapeutic role in apoptosis and fibrosis in I/R injury is unclear. Previous studies have shown that miR-221/222 is involved in pathological cardiovascular mechanisms, including angiogenesis, inflammation, vascular remodeling, fibrosis, cardiac hypertrophy, and apoptosis 12, 13. Bioinformatics analysis showed that PUMA and ETS-1 are candidate targets of miR-221/222. MiR-221/222 induces cell survival by targeting PUMA in glioblastoma 14. ETS-1 upregulation mediates angiotensin II-induced cardiac fibrosis 6. However, the role of miR-221/222 in the heart or during I/R is still unknown, and further research is ongoing. Taking all these factors into consideration, the purpose of this study was to examine whether miR-221/222 participates in the regulation of apoptosis and fibrosis in I/R cardiac injury and H/R-treated cardiomyocytes by targeting PUMA and ETS-1, respectively. Here, we investigated the effects of conditioned medium from adipose-derived stem cells (ADSC-CM) on cardiac apoptosis and fibrosis in I/R-treated mice and H/R-treated H9c2 cells and the underlying mechanisms. The results demonstrated that the cardioprotective effects of ADSC-CM are closely related to reducing apoptosis and fibrosis in I/R-induced cardiac injury through the regulation of miR-221/222.

## Materials and Methods

### *In vivo* myocardial ischemia/reperfusion (I/R) model

The present study used male C57BL/6J wild-type (WT) mice and miR-221/222-knockout (KO) mice aged 8-12 weeks. We generated miR-221/222-KO mice by deleting the X-linked miR-221/222 gene and bred them on a C57BL/6J background for 10 generations. To induce myocardial I/R, the mice were anesthetized with 3% isoflurane. Briefly, the anterior descending branch of the left coronary artery (LAD) was ligated with a 7-0 nylon suture, and a silicone tube (OD 86 mm) was placed 1 mm below the ligation to cause myocardial ischemia. The effectiveness of the occlusion was verified by whitening of the ventricle at the distal end of the ligation. Then, 25 min after the occlusion, 50 μL of ADSC-CM (4 μg/mL) was uniformly injected intramuscularly into five locations in the border area of the anterior wall of the left ventricle. After 30 min of occlusion, the silicone tube was removed to allow blood reperfusion. All mice were anesthetized again after 3 h or 3 d of reperfusion, and the chest was reopened. In the sham operation group, the heart was exposed without LAD ligation. Blood samples and the heart were collected for further analysis. All animal experiments were conducted in accordance with the guidelines for animal care of National Taiwan University (IACUC approval no: 20180426) and complied with the Guide for the Care and Use of Laboratory Animals (NIH publication no. 86-23, revised in 1985). To assess the infarct size, the heart was excised and cut into 1 mm thick cross-sections. The heart sections were then incubated with a 1% triphenyltetrazolium chloride (TTC) solution at 37 °C for 10 min. The infarct area was expressed as the percentage of infarct area (pale) to the total area (red) and measured with ImageJ (NIH, MD, USA). In addition, Evans blue/TTC double staining was used to determine area at risk and infarct area. Briefly, the ligature around LAD was retied and 0.2 mL of 2% Evans blue dye was injected into the left ventricle. The heart was quickly excised and cut into 1 mm thick cross-sections, and then stained with 1% TTC solution at 37 °C for 10 min. The Evans blue-stained area (blue), TTC-stained area (red; area at risk), and TTC-negative staining area (pale; infarct myocardium) were photographed and measured with ImageJ.

### Physiological assessment of cardiac function

The effects of I/R and ADSC-CM on cardiac function were recorded and evaluated by echocardiography. Mice were anesthetized with isoflurane (0.5-1.5% in O_2_) and placed on the rail system to maintain the body temperature (37 °C ± 0.5 °C). Echocardiography was performed with a small animal high-resolution ultrasound system (Prospect, S-Sharp, Taipei, Taiwan) equipped with a 40-MHz single-element transducer. M-mode image was acquired at the level of the papillary muscle of the left ventricle from the long-axis view for evaluation of fractional shortening (FS) and the ejection fraction (EF).

### H9c2 cell culture and* in vitro* hypoxia/reoxygenation (H/R) model

H9c2 cells were originally derived from embryonic rat ventricular cardiomyocytes and were purchased from the American Type Culture Collection (ATCC, VA, USA). Cardiomyocytes were cultured in Dulbecco's modified Eagle's medium (GIBCO, MA, USA) supplemented with 10% fetal bovine serum (Biological Industries, CT, USA) and 1% penicillin (BI). The cultures were maintained in a humid incubator (95% air and 5% CO_2_) at 37 °C. To simulate myocardial I/R injury* in vivo*, H/R was performed on H9c2 cells. H9c2 cells were exposed to hypoxia for 24 h in a hypoxic incubator (5% CO_2_ and 1% O_2_ at 37 °C) in which O_2_ was replaced with N_2_. After hypoxic exposure, the cells were reoxygenated for 12 h at 37 °C in a normoxic incubator with 95% air and 5% CO_2_.

### Preparation of conditioned medium from adipose-derived stem cells (ADSC-CM)

Human adipose-derived stem cells (ADSCs) were purchased from Lonza (Basel, Switzerland). ADSCs were cultured in DMEM containing 20% FBS and penicillin/streptomycin. The cells that attached to the flask were then cultured, and the medium was changed every two days. Cells between the 3rd and 8th passages were used for all experiments. ADSCs were characterized as positive for CD73, CD90, and CD105 and negative for CD34 and CD45 by immunochemistry and flow cytometry. ADSCs have the potential to differentiate into adipocytes, osteoblasts and chondrocytes. To prepare CM from ADSCs, the cells were seeded at 5×10^5^ cells per 10-cm plate. ADSCs reached 80% confluence and were then placed in serum-free medium for 24 h. The medium was then collected for *in vitro* and *in vivo* experiments. The collected CM was concentrated using Amicon Ultra-15 centrifugal filter units (Millipore, MA, USA). The final concentration of the CM was 4 μg/mL. In addition, ADSCs were transfected with miR-221/222 inhibitors for 24 h and the miR-221/222-inhibitors-ADSC-CM was collected to examine the effects of miR-221/222 on the apoptosis and fibrosis of H/R-treated cardiomyocytes.

### Measurement of lactate dehydrogenase

Necrotic cell death was evaluated by measuring LDH activity. The amount of LDH in the serum was determined by the LDH cytotoxicity detection kit (Wako, Osaka, Japan) and then examined by an automatic biochemical analyzer (Hitachi 7070, Tokyo, Japan).

### ROS measurement by dihydroethidium (DHE) staining

DHE is a redox-sensitive probe that is used to measure ROS production. Cryostat sections of cardiac tissues were incubated with 2 μM DHE dye (Beyotime Technology) in the dark at 37 °C for 20 min. The oxidative stress level was examined and observed under a fluorescence microscope. Image-Pro Plus software was used to quantify the red staining, which represented oxidative stress.

### Terminal deoxyribonucleotidyl transferase-mediated dUTP nick-end labeling (TUNEL) analysis

According to the manufacturer's instructions, the *in situ* cell death detection kit (Roche, CA, USA) was used to evaluate apoptotic cells in cardiac sections and cultured cardiomyocytes. Nuclei were stained with DAPI for 10 min. Three slides were prepared for each group. The sections were observed with a confocal microscope. The percentage of TUNEL-positive nuclei relative to the total nuclei was determined in five randomly selected 40× fields on each slide in a blinded manner.

### Western blot

Heart tissues were homogenized and lysed in RIPA buffer (154 mM NaCl, 0.25% sodium deoxycholate, 1% NP-40, 0.8 mM EDTA, and 65.2 mM Tris base) containing a protease inhibitor cocktail (Genestar Biotechnology, Taiwan). Cells were lysed in RIPA buffer (TOOLS, New Taipei City, Taiwan) containing 1% protease inhibitor (Rockford, Illinois, USA) for 1 h at 4 °C. The proteins in tissue and cell lysates were separated by 10% SDS-PAGE (Invitrogen) and transferred to PVDF membranes. The membranes were blocked with TBST containing 5% skim milk for 1 h. Thereafter, the following primary antibodies were used: anti-PUMA (1:2000 dilution, Cell Signaling), anti-phospho-p53 (1:2000 dilution, Cell Signaling), anti-BCL2 (1:2000 dilution, BD, CA, USA), anti-ETS-1 (1:3000 dilution, Abcam), anti-fibronectin (1:3000 dilution, Abcam), anti-collagen 3 (COL3A1; 1:3000 dilution, Proteintech, IL, USA), anti-phospho-p38 MAPK (1:5000 dilution, Cell Signaling), and anti-phospho-NF-κB p65 (1:5000 dilution, Cell Signaling). The membranes were then incubated with horseradish peroxidase-conjugated goat anti-rabbit IgG antibodies (1: 2000 dilution; Sigma, MO, USA) at RT for 1 h. The bound antibodies were detected with Chemiluminescence Plus reagent (Millipore). The images were visualized by the BioSpectrum 815 imaging system (UVP, Upland, CA, USA), and the intensity of each band was quantified using ImageJ software. An antibody against GAPDH (1: 3000 dilution; Santa Cruz Biotechnology) was used as a loading control.

### Histological analysis

All mice were sacrificed, and the hearts were removed. Six hearts were selected from each group for histological analysis. The heart was cut in half by performing a transverse slice between the atrioventricular sulcus and the apex. The sample was fixed in 4% paraformaldehyde, embedded in paraffin, and cut into 5 μm-thick cross-sections. The morphology of the cross-sections was observed with hematoxylin and eosin (HE) staining. The cross-section of the heart was stained with Masson's trichrome (Sigma) to identify collagen deposition, which is shown in blue. Briefly, the heart sections were incubated with Bouin's solution at 56 °C for 15 min and then washed with tap water. The slides were incubated with Weigert's iron hematoxylin solution, Biebrich scarlet-acid fuchsin solution, phosphomolybdic-phosphotungstic acid solution and aniline blue solution. Finally, the slides were treated with a 1% acetic acid solution, dehydrated, and mounted with mounting solution.

### Immunostaining

Heart sections were immersed in 0.01 M sodium citrate for antigen retrieval and treated with 3% H_2_O_2_ for 10 min to remove endogenous peroxidase. The sections were then incubated with 10% normal horse serum (NHS) for 1 h, followed by the primary antibodies anti-PUMA (1:200 dilution, Cell Signaling), anti-ETS-1 (1:200 dilution, Abcam), anti-fibronectin (1:50 dilution, Abcam), and anti-collagen 3 (1:50 dilution, Proteintech) overnight at 4 °C. Then, a second antibody specific to the primary antibody was selected and allowed to react at RT for 90 min. The avidin-biotin-peroxidase-complex was incubated for 1 h. Finally, the tissues were stained with DAB (Sigma) color reaction solution and observed with a light microscope. Additionally, after each designated treatment, the H9c2 cells on the coverslip were fixed in 4% paraformaldehyde for 15 min and then permeabilized with 0.1% Triton X-100 for 1 min at RT. The cells were washed again and then blocked for 1 h with 1% bovine serum albumin (BSA, Novagen) in PBS at RT. The cells were incubated with the previously identified antibodies. After being washed 3 times with PBS, the cells were incubated with anti-rabbit 488 (Invitrogen, A27034) at RT for 1 h. The nuclei were counterstained with DAPI for 5 min, and then the cells were observed and photographed under a fluorescence microscope.

### The assessment of cell death by annexin V/PI double staining

Annexin V-FITC and PI apoptosis detection kits (BD Biosciences, CA, USA) were used to evaluate cell death. H9c2 cells were harvested, resuspended in 100 μL of binding buffer containing 2.5 μL of FITC and 5 μL of PI (100 μg/mL), and incubated in the dark at 4 °C for 15 min. Flow cytometry was used to assess cell death. These graphs are divided into four regions, corresponding to live cells that are negative for both probes (PI-/FITC-; Q3), PI-negative and annexin-positive apoptotic cells (PI-/FITC+; Q1), PI- and annexin-positive late apoptotic cells (PI+/FITC+; Q2), and PI-positive and annexin-negative necrotic cells (PI+/FITC-; Q4).

### RNA isolation and quantitative reverse transcription PCR (RT-qPCR)

Total RNA was isolated from cells or freshly frozen mouse ventricular tissues by using TRIzol reagent (Thermo Fisher, Waltham, USA) according to the manufacturer's protocol. RNA quantification was performed using a NanoDrop ™ 2000 spectrophotometer (Thermo Fisher). For qPCR analysis of miRNA expression, RNA was converted into cDNA by the TaqMan MicroRNA reverse transcription kit (Invitrogen, CA, USA). The expression levels of miR-221 and miR-222 were quantified using commercially available TaqMan MicroRNA assay kits for miR-221 (000524), miR-222 (002276) and RNU6B (001973) and TaqMan™ Universal PCR master mix without UNG in a QuantStudio™ 3 Real-Time PCR System (Applied Biosystems). All reactions were repeated at least three times. The miRNA expression was normalized to the RNU6B control. The fold change in miR-221 and miR-222 expression was quantified by the 2-ΔCt method.

### siRNA transduction

Accell SMARTpool siRNA (Dharmacon, PA, USA) targets and silences ETS-1 or PUMA. A 100 μM siRNA stock solution without RNase was prepared and stored at -20 °C. H9c2 cells were cultured in 6-well plates (Costar, DC, USA) at 70-80% confluence for 24 h, and then the medium was replaced with 1 μM PUMA or ETS-1 siRNA in Lipofectamine 3000 (Thermo Fisher Scientific). The cells were cultured in an incubator at 37 °С for 48 h. The downregulation of ETS-1 and PUMA was confirmed by Western blotting.

### Transient transfection

To manipulate the function of miR-221/222 in H9c2 cells, H9c2 cells were seeded into 6-well plates at a density of 3×10^5^ cells per well and transfected with specific miR-221/222 mimics or a duplex RNA inhibitor (Dharmacon, CO, USA), whose sense sequences were the same as or complementary to miR-221/222, to overexpress or knockdown miR-221/222, respectively. Briefly, according to the manufacturer's protocol, H9c2 cells were grown to 70-80% confluence and transfected with miR-221/222 mimics at a concentration of 100 nM/well using Lipofectamine 3000 reagent (Invitrogen), followed by analysis of ETS-1 and PUMA expression, apoptosis, and fibrosis. Nontargeting sequences were used as negative controls.

Full-length cDNAs encoding rat ETS-1 and PUMA were purchased from Dharmacon. The purified plasmid DNA was diluted in 200 μL of DMEM, mixed with Lipofectamine 3000 (Invitrogen) and incubated at RT for 15 min. H9c2 cells were grown to 60 to 70% confluence in 6-well culture plates. The DNA/Lipofectamine 3000 mixture was added to the cells and incubated at 37 °C for 44 h. H9c2 cells transfected with rat ETS-1 or PUMA were used to analyze cell fibrosis or apoptosis, respectively.

*In vivo* transfection was carried out by TurboFect *in vivo* transfection reagent (Thermo), and miR-221/222 mimics or inhibitors (50 pmole) were prepared at a volume of 50 μL per the manufacturer's instructions. The *in vivo* transfection process followed the protocol of I/R with ADSC-CM injection. The 50 μL mixture of mimics or inhibitors was uniformly injected intramuscularly into five locations in the border area of the anterior wall of the left ventricle.

### Dual-luciferase reporter assay

The wild-type (WT) and mutant (MUT) PUMA 3'-UTR luciferase reporter gene plasmids were produced by Promega (WI, USA). The cells were then cotransfected with miR-NC or miR-221/222 mimics together with the WT or MUT PUMA-3'-UTR reporter plasmid using Lipofectamine 3000 reagent at 37 °C for 48 h. The relative luciferase activity was measured using a dual luciferase assay system (Promega). In addition, the WT and MUT ETS-1 3'-UTR luciferase reporter gene plasmids were produced by Promega. The procedures were similar to those described above.

### Statistical analysis

For each experimental condition, at least three replicates were performed, and the results given are representative of these replicates. The data are expressed as the mean ± standard error of the mean (SEM). Differences between 2 groups were calculated using the Student's t test. ANOVA followed by Dunnett's post hoc test was used for statistical analysis. A p-value less than 0.05 was considered statistically significant.

## Results

### The cardioprotective effects of ADSC-CM are associated with reduced apoptosis and fibrosis in I/R-induced cardiac injury

**Figure 1:**
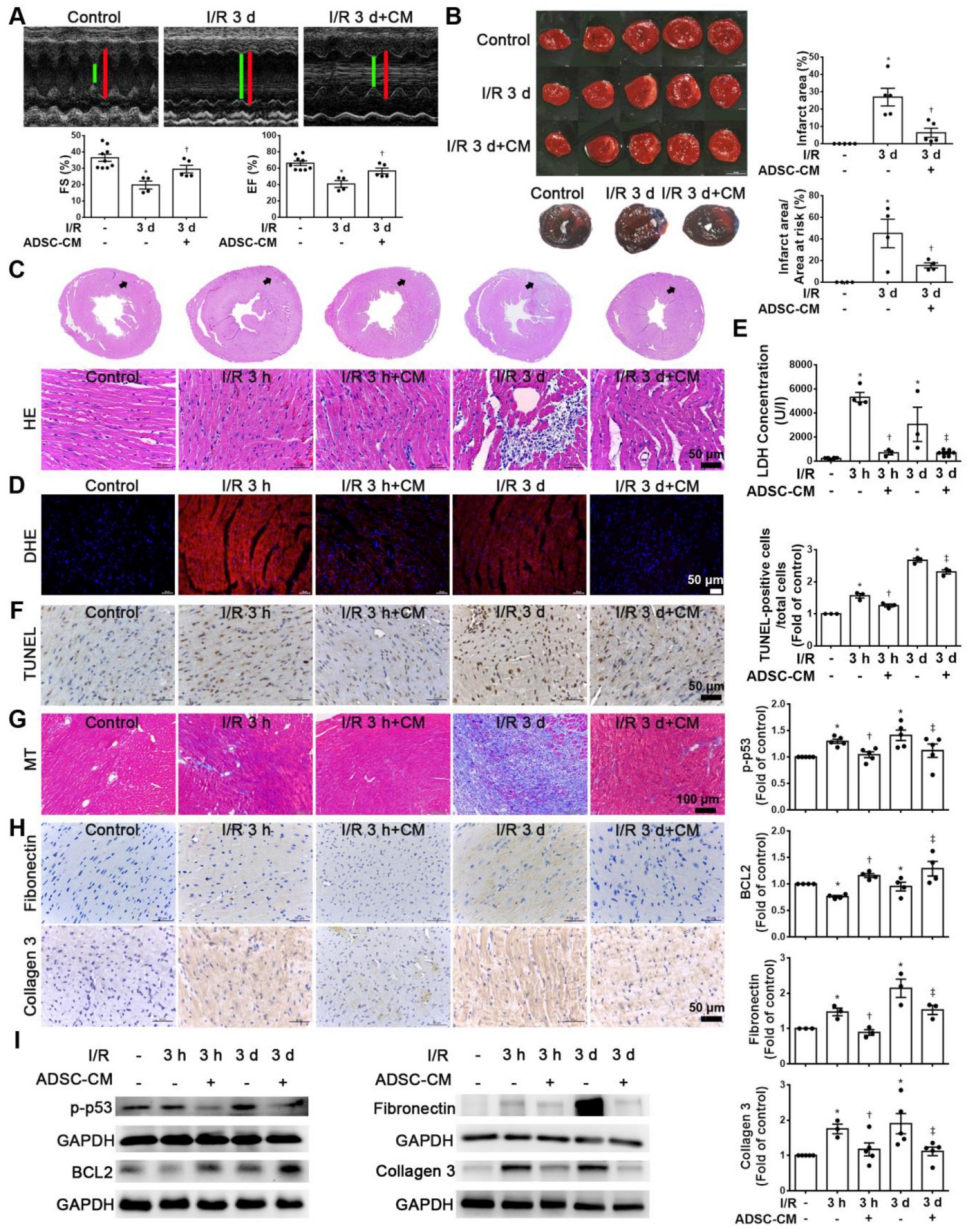
** ADSC-CM treatment reduced I/R-induced cardiac apoptosis and fibrosis.** The left anterior descending coronary artery (LAD) of male C57/B6J mice was ligated for 30 min and then reperfused for 3 h (I/R 3 h) or 3 d (I/R 3 d). In ADSC-CM-treated animals, ADSC-CM (4 μg/mL) was injected into the anterior wall of the left ventricle. Sham-operated animals underwent the same procedure without occlusion of the LAD. (A) Representative echocardiogram from the control, I/R 3 d, and I/R 3 d+CM mice. Quantitative data of the fractional shortening (FS) and the ejection fraction (EF). (B) The infarct area was determined by TTC staining. The ischemic area showed pale and viable myocardium showed red. Infarct area was quantified as a percentage of total slice area. Infarct area and area at risk quantification by Evans blue and TTC double staining. Graphic representation of the infarct size expressed as percentage of infarct area over area at risk. (C)The morphology of cardiac sections was observed by HE staining (scale bar = 50 μm). (D) Measurement of intracellular ROS was performed by DHE staining (scale bar = 50 μm). (E) LDH levels in serum were measured by an LDH cytotoxicity detection kit. Myocardial apoptosis was determined and quantified by TUNEL assay (TUNEL-positive cells: brown; nuclei: blue; scale bar = 50 μm). (F) The presence of collagen deposition was evaluated by Masson's trichrome (MT) staining (scale bar =100 μm). (G) The expression of fibronectin and collagen 3 was examined by immunohistochemistry (scale bar = 50 μm). (H) The expression levels of p-p53, BCL2, fibronectin, and collagen 3 expression were measured by Western blotting. The data are expressed as the mean ± SEM (n = 3-5). *P < 0.05 vs. control, ^†^P < 0.05 vs. the I/R 3 h group, ^‡^P< 0.05 vs. the I/R 3 d group.

Echocardiographic analysis was used to examine the effects of ADSC-CM on the cardiac function. M‐mode images of three groups were shown in Figure 1A. Compared with the control group, I/R significantly reduced fractional shortening (FS) and ejection fraction (EF). In contrast, ADSC-CM treatment significantly increased FS and EF. Meanwhile, TTC staining was performed to evaluate the infarct area (Figure 1B). The area of myocardial infarction in I/R mice at 3 days was 26.9±5.1%. But in mice treated with ADSC-CM, it was significantly smaller (6.3±2.6%). Compared with the control group, the I/R group showed a significant myocardial infarction area by Evans blue and TTC double staining, while pretreatment with ADSC-CM reduced the myocardial infarction area. In addition, we examined the recruitment of inflammatory cells in the infarcted area at 3 h and 3 d after myocardial infarction (I/R 3 h and I/R 3 d groups, respectively) by H&E staining. Inflammatory cell infiltration was obviously observed in the I/R 3 d group but not the I/R 3 h group. Monocyte infiltration into the infarcted area was reduced by ADSC-CM treatment (Figure 1C). Oxidative stress plays an important role in cardiac damage caused by I/R. Therefore, we examined the effect of ADSC-CM on ROS production. As shown in Figure 1D, DHE-positive cells were clearly observed after I/R induction (I/R 3 h and I/R 3 d groups). ADSC-CM greatly reduced the generation of ROS. LDH release is an indicator of cell damage. As shown in Figure 1E, the release of LDH in plasma was significantly increased in the I/R group. However, ADSC-CM significantly reduced the release of LDH. TUNEL analysis showed that I/R induced a significant increase in apoptosis, while ADSC-CM reduced the level of apoptosis (Figure 1F). In addition, I/R increased collagen deposition, as observed by Masson's trichrome staining, while ADSC-CM reduced collagen deposition (Figure 1G). The immunohistochemistry results showed that I/R induced the expression of fibronectin and collagen 3 (fibrosis markers), while ADSC-CM reduced the expression of these markers (Figure 1H). To further investigate the molecular mechanisms involved, the protein levels of apoptosis markers such as p-p53 and BCL2 and fibrotic markers such as fibronectin and collagen 3 were examined. As shown in Figure 1I, I/R significantly increased the expression of p-p53, fibronectin and collagen 3, while ADSC-CM treatment significantly reduced the expression of these factors.

### ADSC-CM protected against myocardial I/R injury in mice through miR-221/222

**Figure 2:**
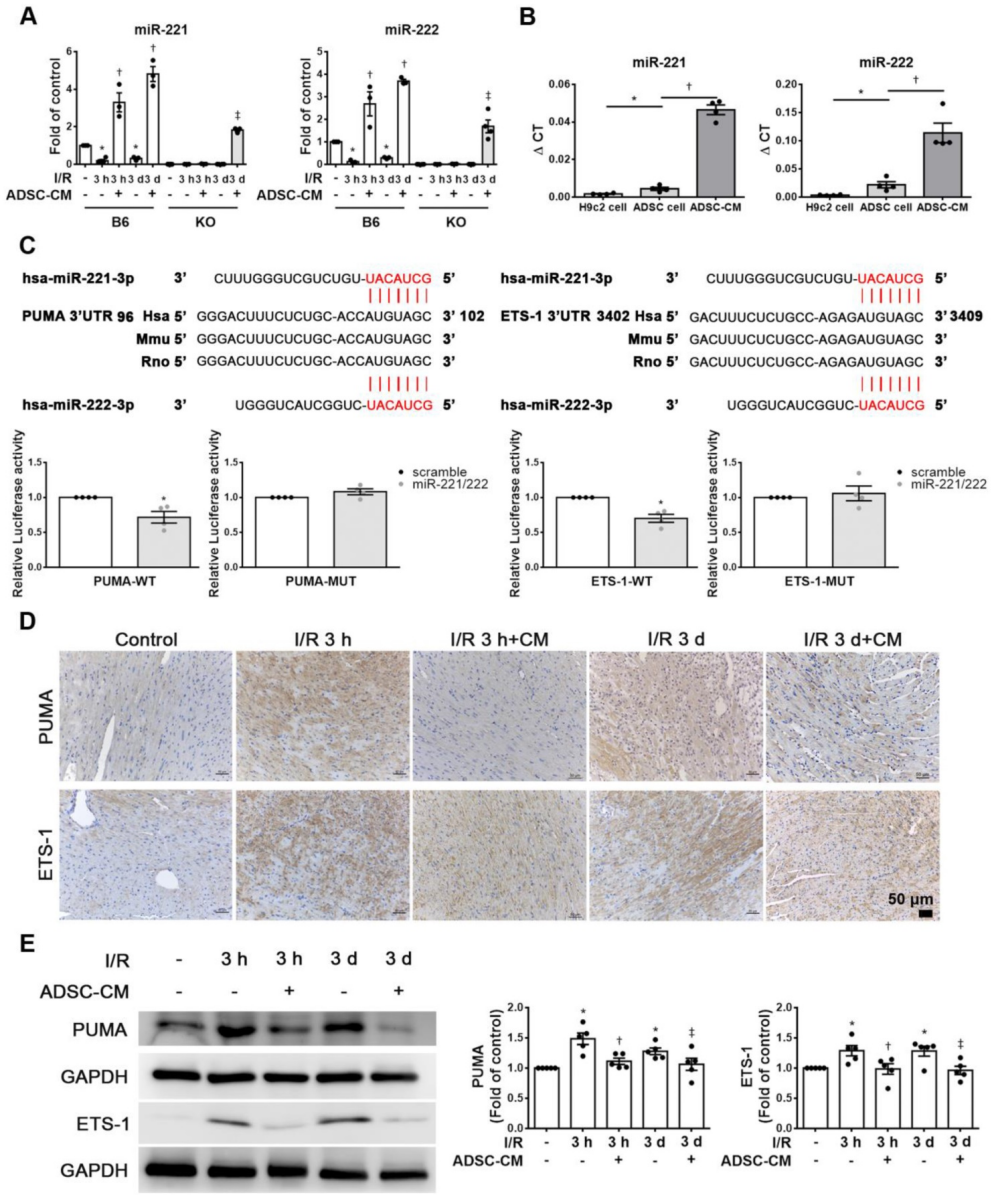
** ADSC-CM protected against myocardial I/R injury in mice through miR-221/222.** (A) The levels of miR-221/222 were measured by RT-qPCR. (B) ADSC-CM contains a large amount of miR-221/222. (C) Nucleotide resolution of the predicted miR-221/222 binding sequence in PUMA and ETS-1. Luciferase activity in H9c2 cells cotransfected with PUMA-WT or PUMA-MUT, ETS-1-WT or ETS-1-MUT and miR-NC mimic or miR-221/222 mimics. (D). The expression of PUMA and ETS-1 in cardiac tissues was determined by immunohistochemistry (scale bar = 50 μm). (E) Western blot analysis of PUMA and ETS-1 expression. The data are expressed as the mean ± SEM (n = 3-5). ^*^P < 0.05 vs. control, ^†^P < 0.05 vs. the I/R 3 h group, ^‡^P< 0.05 vs. the I/R 3 d group or KO control.

The expression of miR-221/222 in I/R-injured hearts was significantly downregulated, as shown by RT-qPCR. After injecting ADSC-CM into the myocardium, the expression of miR-221/222 was significantly increased (Figure 2A). Importantly, ADSC-CM contained a large amount of miR-221/222 compared to H9c2 cells and ADSC lysates (Figure 2B). The miR-221/222 target sites in the 3' untranslated regions (3'-UTRs) of the PUMA and ETS-1 genes were predicted using the "miRanda" target prediction program. Specifically, the 3'-UTRs of PUMA and ETS-1 contain binding sites for miR-221/222. To confirm this prediction, we conducted a PUMA and ETS-1 luciferase assay with constructs containing the miR-221/222 binding sequences of the PUMA and ETS-1, and miR-221/222 mimics resulted in reduced luciferase activity. The relative luciferase activity of PUMA-MUT and ETS-1-MUT were not significantly different (Figure 2C). As shown in Figure 2D, in the control group, PUMA and ETS-1 expression was not observed by immunohistochemistry, while in the I/R treatment group, strong staining was observed in cardiac tissues. In contrast, the administration of ADSC-CM resulted in weakened PUMA and ETS-1 staining in I/R-treated animals. Similarly, Western blotting was used to confirm these results (Figure 2E). Western blot analysis showed that the protein levels of PUMA and ETS-1 were increased in response to I/R induction, while ADSC-CM decreased the expression of these factors.

### ADSC-CM reduced apoptosis and fibrosis in H9c2 cells stimulated with hypoxia/reoxygenation (H/R)

**Figure 3:**
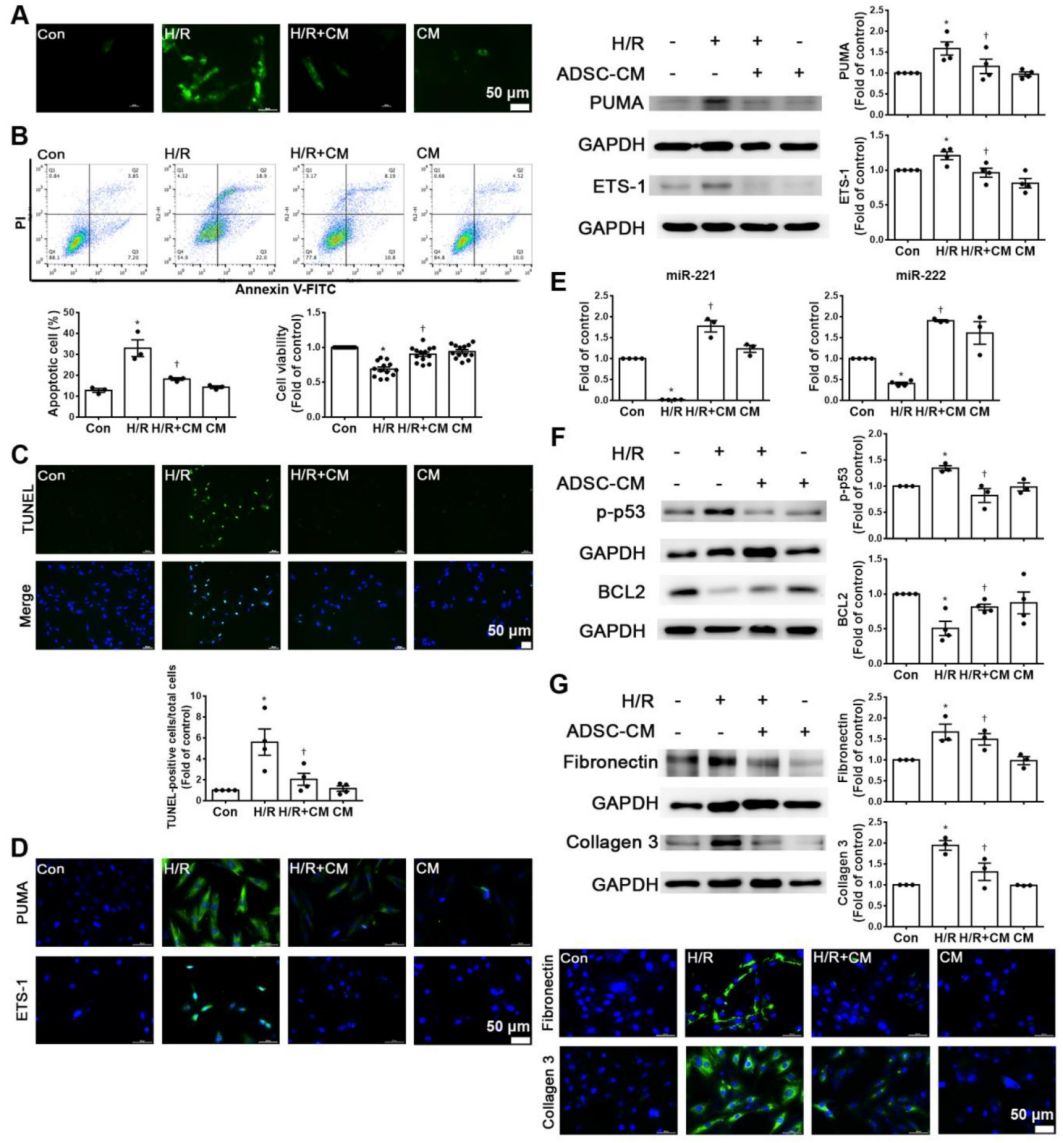
** ADSC-CM attenuated hypoxia/reoxygenation (H/R)-induced apoptosis and fibrosis in H9c2 cells.** Cardiomyocytes were pretreated with or without ADSC-CM for 4 h and then exposed to hypoxia for 24 h. The cells were then exposed to normoxia for another 12 h. (A) DCFH-DA staining was used to examine cytoplasmic H_2_O_2_ by fluorescence microscopy (scale bar = 50 μm). (B) Apoptosis and cell viability were evaluated by flow cytometry with annexin V/PI staining and MTT assays. (C) Apoptosis was examined by TUNEL assay (green: TUNEL-positive nuclei; blue: DAPI-positive nuclei; scale bar = 50 μm). (D) The expression levels of PUMA and ETS-1 were examined by immunofluorescent staining and Western blot (scale bar = 50 μm). (E) MiR-221/222 levels were measured by RT-qPCR. (F) The levels of apoptosis markers (p-p53 and BCL2) were examined by Western blot. (G) The levels of fibrosis markers (fibronectin and collagen 3) were examined by Western blot and immunofluorescent staining (scale bar = 50 μm). The data are expressed as the mean ± SEM (n = 3-4). ^*^P < 0.05 vs. control, ^†^P < 0.05 vs. H/R.

Hypoxia/reoxygenation (H/R) is an important factor in the induction of apoptosis and fibrosis 15, 16. H9c2 cells were pretreated with or without ADSC-CM for 4 h and then place in serum-free medium. H9c2 cells were incubated at 37 °C for 24 h under 1% O_2_ to induce hypoxia. After that, the cells were placed in a normoxic incubator for 12 h. H/R increased ROS production, while ADSC-CM significantly reduced ROS production, as shown by DCFH-DA staining (Figure 3A). H/R increased apoptosis and decreased cell viability compared to those of control cells, as shown by flow cytometry and MTT analysis (Figure 3B). Apoptosis was further assessed by TUNEL assay. Treatment with ADSC-CM significantly decreased H/R-induced apoptosis (Figure 3C). H/R induced the expression of PUMA and ETS-1, and ADSC-CM treatment reduced the expression of these factors, as shown by Western blotting and immunofluorescent staining (Figure 3D). Because ADSC-CM increased miR-221/222 expression in I/R-treated mice, we examined whether miR-221/222 could protect cardiomyocytes from H/R-induced apoptosis and fibrosis.

The expression of miR-221/222 was significantly downregulated in the H/R treatment group compared with the control group, as shown by RT-qPCR, while ADSC-CM increased miR-221/222 expression (Figure 3E). To further investigate the molecular mechanisms involved, the protein levels of apoptosis and fibrosis markers (such as p-p53, BCL2, fibronectin and collagen 3) were examined. As shown in Figure 3F, H/R significantly increased the expression level of p-p53 and decreased the expression level of BCL2, while ADSC-CM reversed these effects. In addition, H/R significantly increased the expression of fibronectin and collagen 3, while ADSC-CM administration markedly reduced the expression of these factors, as shown by Western blotting and immunofluorescent staining (Figure 3G). Taken together, these data indicate that miR-221/222 is an effective regulator of cardiac apoptosis and fibrosis. The administration of ADSC-CM resulted in decreases in H/R-induced apoptosis and fibrosis through miR-221/222.

### MiR-221/222 associated with ADSC-CM reduced apoptosis and fibrosis of H/R-treated H9c2 cells

**Figure 4:**
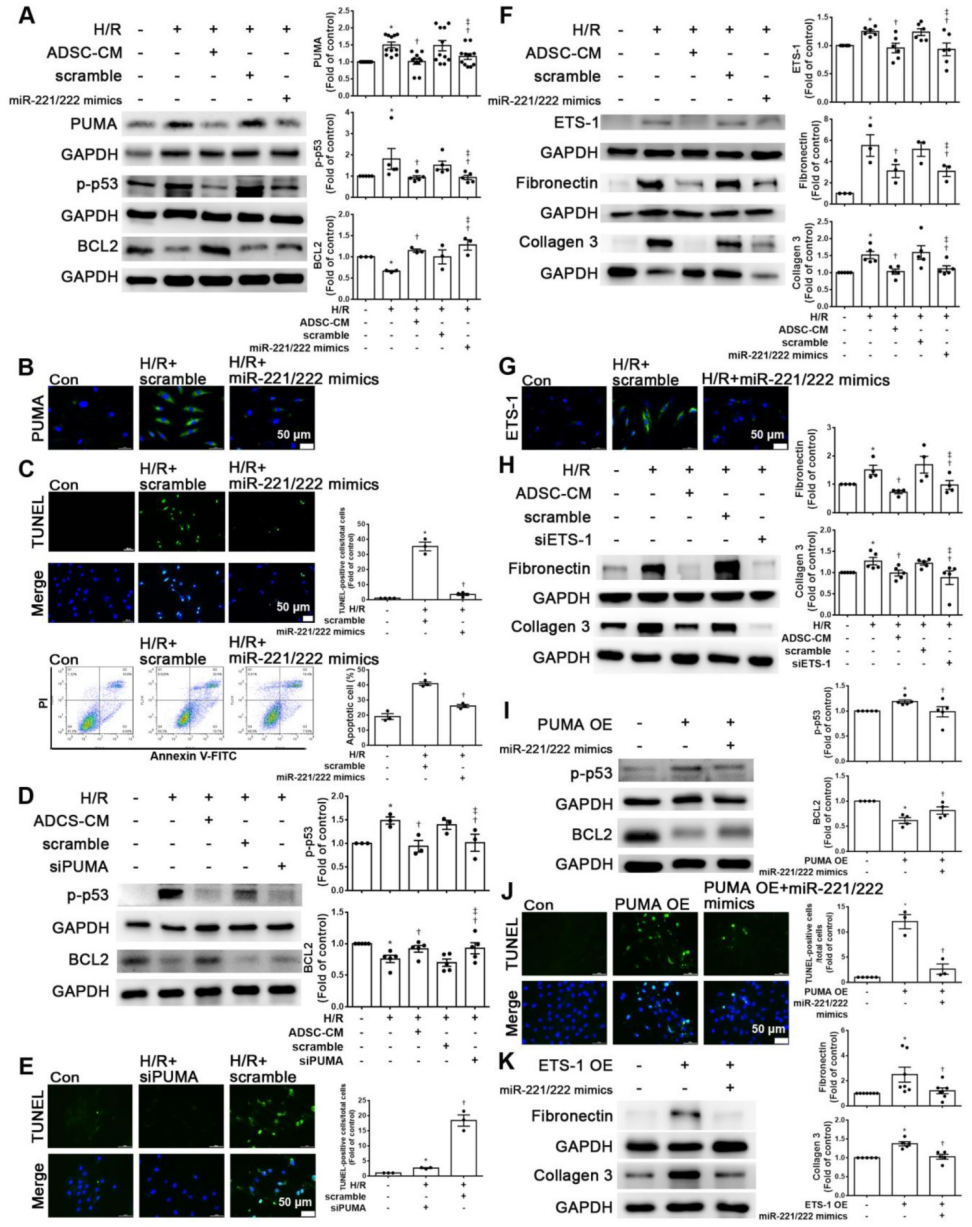

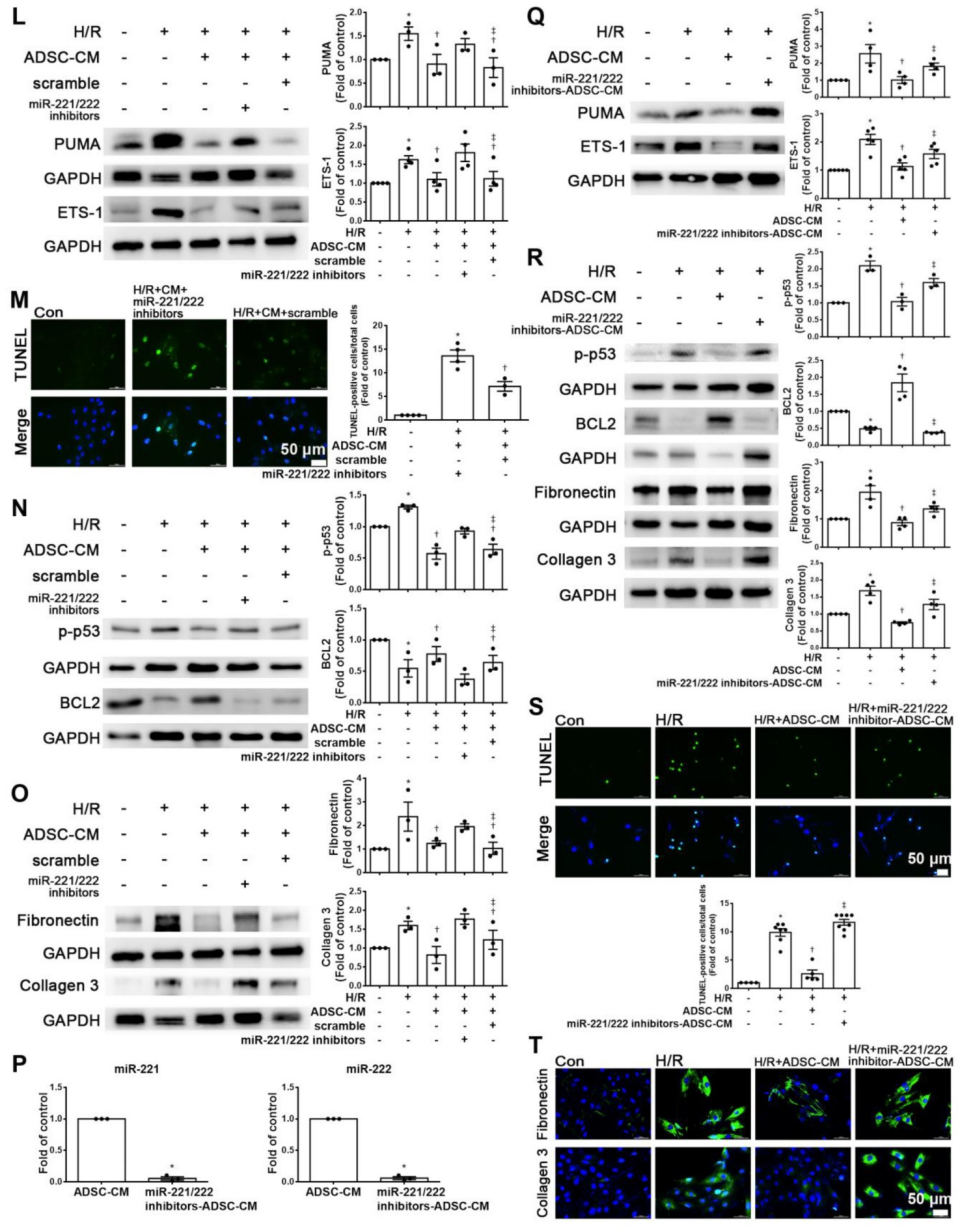
** MiR-221/222 was involved in the ADSC-CM-mediated reduction in H/R-induced apoptosis and fibrosis in H9c2 cells.** (A) Western blot analysis of PUMA, p-p53, and BCL2 expression in cells transfected with miR-221/222 mimics. (B) PUMA expression was examined by immunofluorescent staining (scale bar = 50 μm). (C) Transfection with miR-221/222 mimics significantly decreased H/R-induced apoptosis, as examined by TUNEL assay and flow cytometry using annexin V/PI (scale bar = 50 μm). (D) Knockdown of PUMA in H9c2 cells exposed to H/R increased BCL2 expression and decreased p-p53, as shown by Western blot. (E) The number of apoptotic H9c2 cells after siPUMA transfection and H/R treatment was examined by TUNEL assay (scale bar = 50 μm). (F) Transfection of miR-221/222 mimics significantly reduced ETS-1, fibronectin and collagen 3 expression in H/R-treated H9c2 cells, as shown by Western blot. (G) ETS-1 expression was examined by immunofluorescent staining (scale bar = 50 μm). (H) Knockdown of ETS-1 in H9c2 cells exposed to H/R reduced fibronectin and collagen 3 expression, as shown by Western blot. (I) The overexpression of PUMA significantly decreased BCL2 expression and increased p-p53 expression, as shown by Western blot. (J) PUMA-overexpressing cells transfected with miR-221/222 mimics decreased apoptosis, as shown by TUNEL assay (scale bar = 50 μm). (K) ETS-1-overexpressing cells transfected with miR-221/222 mimics decreased fibronectin and collagen 3 expression, as shown by Western blot. (L) ADSC-CM-treated cells treated by H/R and transfected with miR-221/222 inhibitors increased PUMA and ETS-1 expression, as shown by Western blot. (M) ADSC-CM-treated cells exposed to H/R and transfected with miR-221/222 inhibitors increased the number of apoptotic cells, as shown by TUNEL assay (scale bar = 50 μm). (N, O) Western blot analysis showed that the miR-221/222 inhibitors increased the protein levels of p-p53, fibronectin and collagen 3. (P) ADSCs were transfected with miR-221/222 inhibitors and then the conditioned media were collected. MiR-221/222 levels were measured by RT-qPCR. (Q, R) MiR-221/222 inhibitors-ADSC-CM was used to examine the effects of miR-221/222 on the expression of PUMA, ETS-1, p-p53, BCL2, fibronectin, and collagen 3 in H/R-treated H9c2 cells. (S) MiR-221/222 inhibitors-ADSC-CM was used to examine the effects of miR-221/222 on cell apoptosis in H/R-treated H9c2 cells, as shown by TUNEL assay (scale bar = 50 μm). (T) MiR-221/222 inhibitors-ADSC-CM was used to examine the effects of miR-221/222 on the expression of fibronectin and collagen 3 by immunofluorescent staining (scale bar = 50 μm). The data are expressed as the mean ± SEM (n = 3-7). ^*^P < 0.05 vs. control, ^†^P < 0.05 vs. H/R, PUMA-OE, ETS-1-OE, ^‡^P< 0.05 vs. H/R+scramble, H/R+miR221/222 mimics, H/R+ADSC-CM.

To clarify the effects of miR-221/222 on apoptosis and fibrosis, the expression levels of the apoptotic and fibrotic markers PUMA and ETS-1, respectively, in cardiomyocytes transfected with miR-221/222 mimics were measured by Western blot analysis. MiR-221/222 mimics reduced PUMA and p-p53 expression in H/R-treated cardiomyocytes and increased BCL2 expression (Figure 4A). The immunofluorescent staining of PUMA was consistent with the Western blot results (Figure 4B). The transfection of miR-221/222 mimics also significantly reduced H/R -induced apoptosis, as shown by TUNEL analysis and flow cytometry (Figure 4C). Knockdown of PUMA in H9c2 cells exposed to H/R reduced p-p53 and increased BCL2, modulating apoptosis (Figure 4D). TUNEL analysis also confirmed these results (Figure 4E). In addition, miR-221/222 mimics significantly reduced ETS-1 expression in H/R-treated cardiomyocytes, and transfection with miR-221/222 mimics also significantly decreased H/R-induced fibronectin and collagen 3 expression, as shown by Western blot (Figure 4F). The results of immunofluorescent staining of ETS-1 were consistent with the Western blot results (Figure 4G). Knocking down ETS-1 in H9c2 cells exposed to H/R reduced the expression of fibronectin and collagen 3 (Figure 4H).

Enhanced PUMA expression increased p-p53 and decreased BCL2 expression, while transfection of miR-221/222 mimics reversed the effect of PUMA overexpression, as shown by Western blotting (Figure 4I). The TUNEL assay also confirmed this result (Figure 4J). In addition, enhanced ETS-1 expression increased the expression of fibronectin and collagen 3, while transfection of miR-221/222 mimics reversed the effect of ETS-1 overexpression, as shown by Western blot (Figure 4K). Therefore, these results indicate that PUMA and ETS-1 are involved in apoptosis and fibrosis mediated by oxidative stress through the regulation of miR-221/222. Western blot analysis showed that the miR-221/222 inhibitors increased the protein levels of PUMA and ETS-1 in H/R-treated cells treated with ADSC-CM (Figure 4L). Transfection with miR-221/222 inhibitors increased apoptosis compared with that of the H/R+ ADSC-CM group (Figure 4M). The miR-221/222 inhibitors reduced the effects of ADSC-CM on the expression of p-p53, BCL2, fibronectin, and collagen 3 in H/R-treated cells, as shown by Western blot (Figure 4N-O). ADSCs were transfected with miR-221/222 inhibitors, and then the conditioned media were collected. The levels of miR-221/222 were significantly decreased in miR-221/222 inhibitors-ADSC-CM when compared with those in the ADSC-CM by RT-qPCR (Figure 4P). These conditioned media were used to examine the effects of miR-221/222 on apoptosis and fibrosis of H/R-treated H9c2 cells. The miR-221/222 inhibitors-ADSC-CM increased the levels of PUMA and ETS-1 expression when compared with H/R+ADSC-CM-treated cardiomyocytes (Figure 4Q). The miR-221/222 inhibitors-ADSC-CM also increased p-p53 and decreased BCL2 expression (Figure 4R). Similarly, TUNEL assay was used to confirm these results (Figure 4S). In addition, through Western blot and immunofluorescent staining, ADSC-CM significantly reduced the expression of fibronectin and collagen 3 in H/R-treated cells, while miR-221/222 inhibitors-ADSC-CM increased their expression (Figure 4R, T).

### ADSC-CM reduced apoptosis and fibrosis in H/R-treated H9c2 cells via the p38 and NF-κB pathways

**Figure 5:**
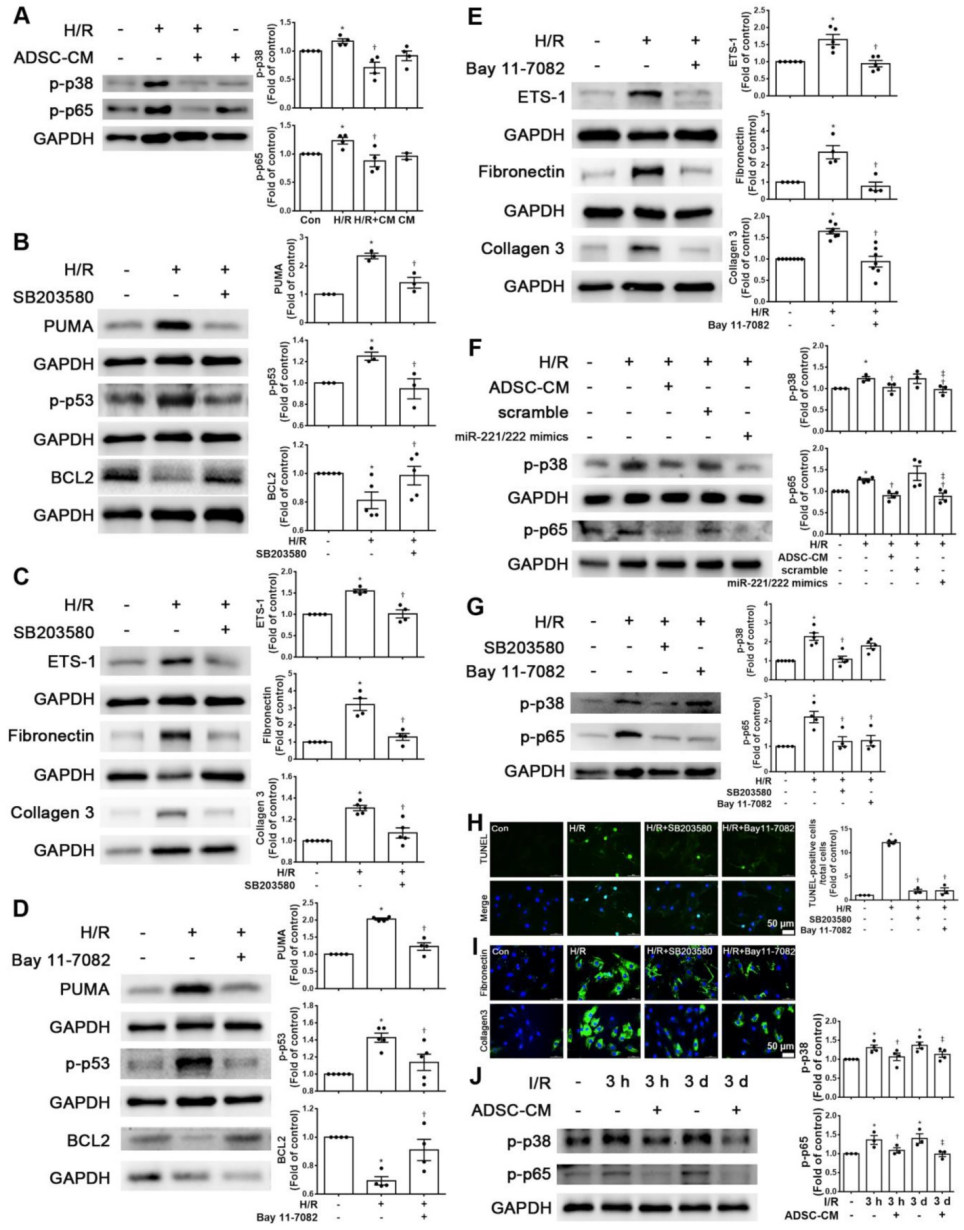
** ADSC-CM reduced apoptosis and fibrosis in H/R-treated H9c2 cells through the p-p38/NFκB p65 pathway.** (A) Cardiomyocytes were pretreated with or without ADSC-CM for 4 h and then exposed to hypoxia for 24 h. The cells were then exposed to normoxia for another 12 h. The phosphorylation levels of p38 and NFκB p65 were examined by Western blot. (B, C) Cells were pretreated with or without SB203580 (10 μM, p38 inhibitor) for 1 h and then induced with H/R. The expression levels of PUMA, p-p53, BCL2, ETS-1, fibronectin, and collagen 3 were examined by Western blot. SB203580 reduced the expression of PUMA, p-p53, ETS-1, fibronectin, and collagen 3, while increasing BCL2 expression in H/R-treated H9c2 cells. (D, E) Cells were pretreated with or without Bay 11-7082 (2.5 μM, NFκB inhibitor) for 1 h and then induced with H/R. Bay 11-7082 reduced the expression of PUMA and p-p53 and increased the expression of BCL2. Bay 11-7082 also significantly reduced H/R-induced ETS-1, fibronectin, and collagen 3 expression in H9c2 cells. (F) The transfection of miR-221/222 mimics significantly ameliorated H/R-reduced p38 and p65 phosphorylation, as shown by Western blot. (G) The p38 inhibitor reduced the phosphorylation of NFκB, while the NF-κB inhibitor did not affect p38 phosphorylation, as shown by Western blot. (H, I) Treatment with the p38 inhibitor or with the NFκB inhibitor decreased apoptosis and fibrosis, as shown by TUNEL assay and immunofluorescent staining, respectively. (scale bar = 50 μm). (J) I/R significantly increased p-p38 and p-p65 compared to those of the control mice, as shown by Western blot, while ADSC-CM reversed these effects *in vivo*. The data are expressed as the mean ± SEM (n = 3-7). ^*^P < 0.05 vs. control, ^†^P < 0.05 vs. H/R, I/R, ^‡^P< 0.05 vs. H/R+scramble, H/R+miR221/222 mimics.

Increasing evidence has shown that p-p38 and NF-kB proteins play critical roles in H/R-induced injury 17, 18. We first examined the phosphorylation levels of p38 and NF-kB p65 in H9c2 cells stimulated with H/R. The phosphorylation levels of p38 and p65 were increased in H/R-treated H9c2 cells compared with control cells, while ADSC-CM treatment reduced the phosphorylation levels, as shown by Western blot (Figure 5A). To determine whether the effect of ADSC-CM on apoptosis and fibrosis in H/R-treated H9c2 cells requires the p38 pathway, the p38 inhibitor SB203580 was used during H/R induction. SB203580 reduced the expression of PUMA, p-p53, ETS-1, fibronectin, and collagen 3 in H/R-treated H9c2 cells while increasing BCL2 expression (Figure 5B-C). To confirm the role of NF-κB p65 in the antiapoptotic and antifibrotic effects on ADSC-treated H9c2 cells, the NF-κB inhibitor Bay 11-7082 was used and reduced the expression of PUMA and p-p53 and increased the expression of BCL2 in H/R-treated H9c2 cells (Figure 5D). In addition, Bay 11-7082 reduced H/R-induced ETS-1, fibronectin, and collagen 3 expression in H9c2 cells (Figure 5E). Transfection of the miR-221/222 mimics significantly reduced the H/R- increased p38 and p65 phosphorylation (Figure 5F). Furthermore, SB203580 reduced NFκB phosphorylation, while Bay 11-7082 treatment had no significant effect on p38 phosphorylation (Figure 5G). SB203580 and Bay 11-7082 treatment significantly reduced apoptosis, as shown by TUNEL assay (Figure 5H). Consistent with the Western blot results, SB203580 and Bay 11-7082 reduced the expression of fibronectin and collagen 3, as shown by immunofluorescent staining (Figure 5I). In addition, I/R significantly reduced p-p38 and p-p65 compared to those of control mice, as shown by Western blot, while ADSC-CM reversed these effects *in vivo* (Figure 5J). Taken together, these findings indicate that ADSC-CM prevents cardiac I/R injury through the p38/NFκB p65 pathway.

### ADSC-CM protected against myocardial I/R injury in miR-221/222 KO mice

**Figure 6:**
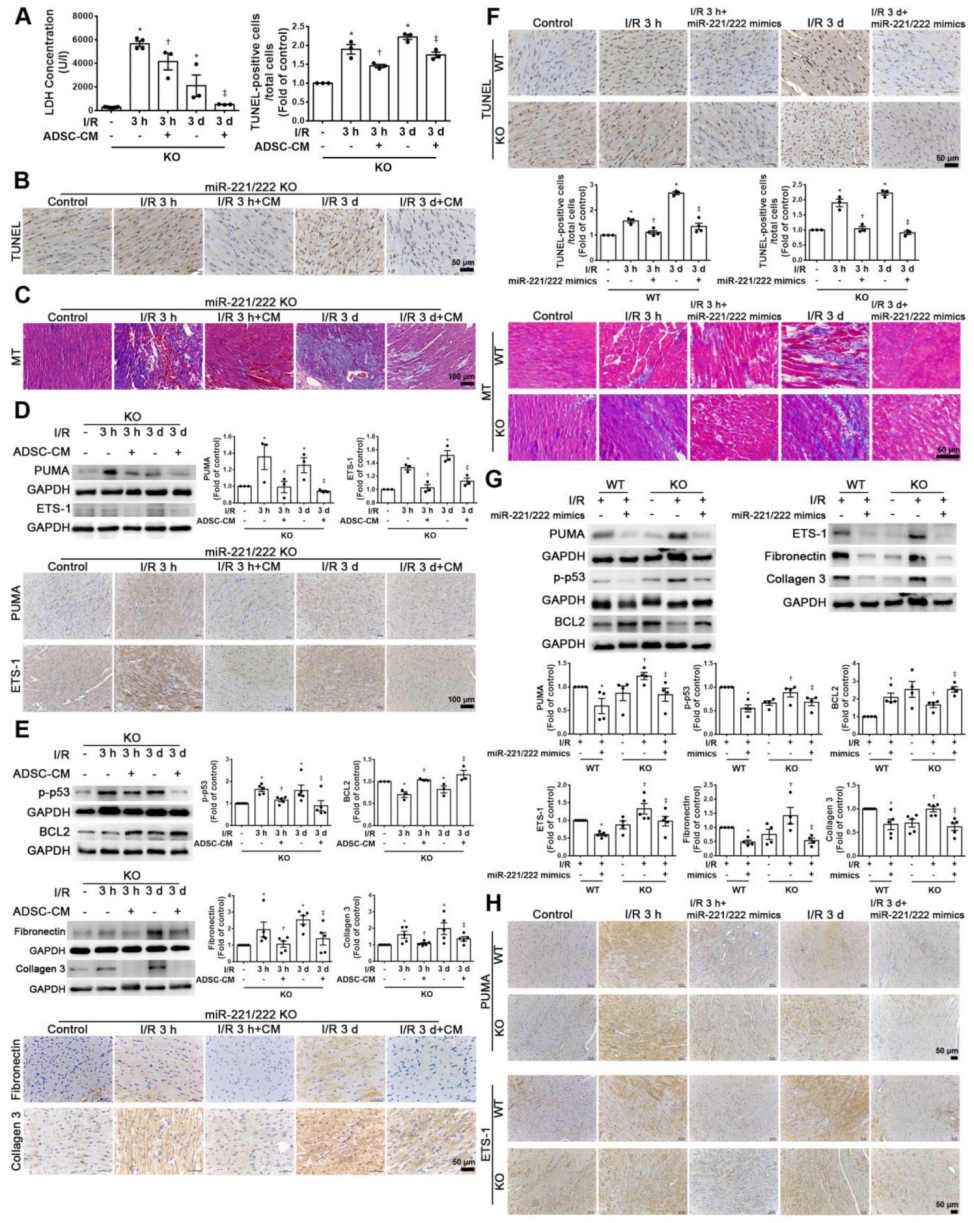

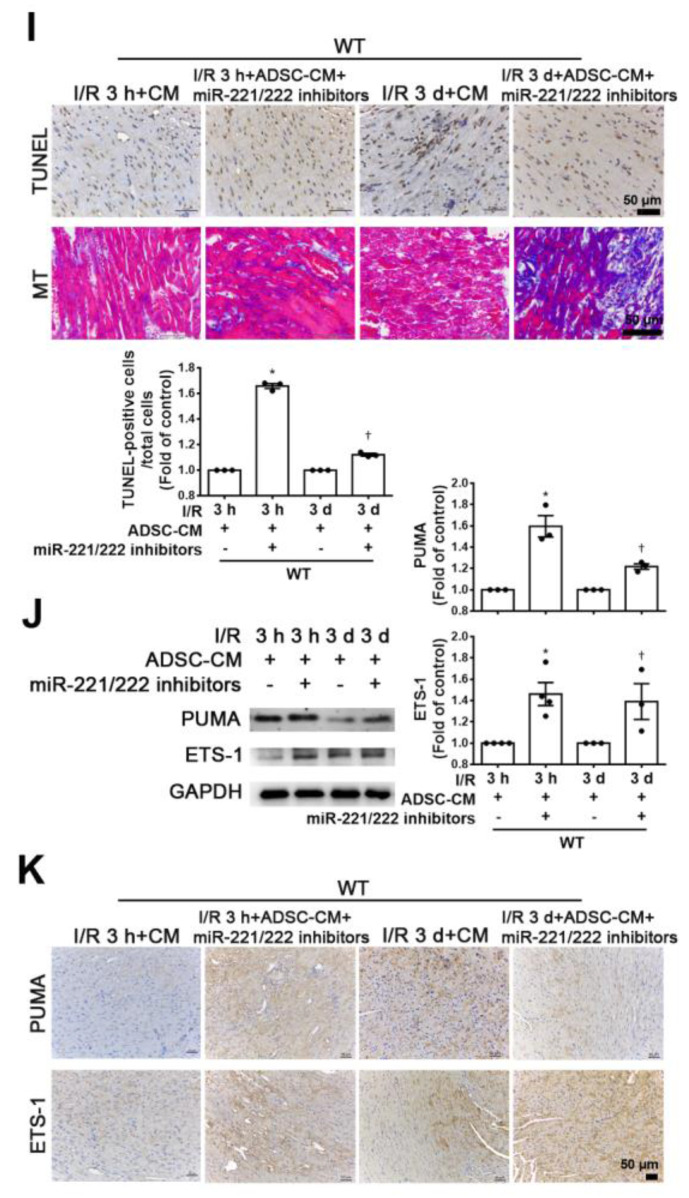
** ADSC-CM ameliorated myocardial I/R injury in miR-221/222 KO mice.** The LAD of miR-221/222 KO mice was occluded for 30 min and then reperfused for 3 h or 3 d. In ADSC-CM-treated animals, ADSC-CM (4 μg/mL) was injected into the anterior wall of the left ventricle. (A) ADSC-CM significantly inhibited I/R-increased LDH levels. (B, C) The levels of apoptosis and fibrosis were examined by TUNEL assay and Masson's trichrome (MT) staining, respectively (scale bar = 50 μm; 100 μm). (D) The protein levels of PUMA and ETS-1 were evaluated by Western blot analysis and immunohistochemistry (scale bar = 100 μm). (E) The expression levels of p-p53, BCL2, fibronectin and collagen 3 were examined by Western blot and immunohistochemistry (scale bar = 50 μm). (F) The effects of the* in vivo* transfection of miR-221/222 mimics on apoptosis and fibrosis were examined by TUNEL assay and Masson's trichrome staining, respectively, in normal mice and miR-221/222 KO mice. (scale bar = 50 μm). (G) The effect of the* in vivo* transfection of miR-221/222 mimics treatment on the expression of PUMA, p-p53, BCL2, ETS-1, fibronectin and collagen 3 was measured by Western blot. (H) The effects of the* in vivo* transfection of miR-221/222 mimics treatment on the expression of PUMA and ETS-1 were examined by immunohistochemistry (scale bar = 50 μm). (I) The effects of the *in vivo* transfection of miR-221/222 inhibitors on apoptosis and fibrosis were examined by TUNEL assay and Masson's trichrome staining, respectively (scale bar = 50 μm). (J, K) The expression of PUMA and ETS-1 in WT mice transfected with miR-221/222 inhibitors was evaluated by Western blot and immunohistochemistry. The data are expressed as the mean ± SEM (n = 3-5). ^*^P < 0.05 vs. control, ^†^P < 0.05 vs. the I/R 3 h or KO control group, ^‡^P< 0.05 vs. the I/R 3 d or KO I/R group.

Compared with that of the sham operation group, the serum LDH activity was significantly increased in the I/R group of miR-221/222 KO mice. However, the addition of ADSC-CM significantly reduced LDH levels after I/R induction in miR-221/222 KO mice (Figure 6A). In addition, after I/R induction of miR-221/222 KO mice, TUNEL analysis and Masson's trichrome staining showed that apoptosis and fibrosis were significantly increased, while ADSC-CM treatment reduced apoptosis and fibrosis (Figure 6B-C). After I/R, the expression of PUMA and ETS-1 in miR-221/222 KO mice was significantly increased, as shown by Western blot and immunohistochemistry, and these effects were reduced by ADSC-CM administration (Figure 6D). In addition, the levels of p-p53, fibronectin and collagen 3 expression were upregulated in the I/R group, while ADSC-CM decreased the expression (Figure 6E). Consistent with the results of ADSC-CM treatment, compared with the I/R group, the group transfected with miR-221/222 mimics exhibited significantly reduced apoptosis and fibrosis, as shown by TUNEL analysis and Masson's trichrome staining, respectively (Figure 6F). The protein expression of BCL2 in the I/R group was reduced, while* in vivo* transfection of miR-221/222 mimics increased BCL2 expression (Figure 6G). Moreover, the expression levels of p-p53, fibronectin and collagen 3 were upregulated in the I/R group, while *in vivo* transfection of miR-221/222 mimics reduced the expression of these factors. In addition, the PUMA and ETS-1 levels of the I/R group increased, while the transfection of miR-221/222 mimics reduced the expression of these factors (Figure 6G). Similarly, these results were confirmed by immunohistochemistry (Figure 6H). In contrast, the I/R+ADSC-CM+miR-221/222 inhibitor group did not exhibit decreased apoptosis and fibrosis compared to the I/R group (Figure 6I). The expression of PUMA and ETS-1 was upregulated in the I/R group and was not affected in the I/R+ADSC-CM+miR-221/222 inhibitor group (Figure 6J). Similarly, these data were confirmed by immunohistochemistry (Figure 6K). These findings strongly support that ADSC-CM prevents myocardial I/R injury through miR-221/222 *in vivo*.

## Discussion

In this study, we demonstrated reductions in miR-221/222 expression levels in I/R-treated hearts and H/R-treated H9c2 cells. In addition, ADSC-CM containing large amounts of miR-221/222 can reduce cardiac apoptosis and fibrosis. MiR-221/222 can target and regulate PUMA or ETS-1 expression levels. Furthermore, miR-221/222 regulates the PUMA/p53/BCL2 pathway to mediate cardiomyocyte apoptosis and the ETS-1/fibronectin/collagen 3 pathway to mediate fibrosis through the p38/NF-κB pathway.

I/R injury leads to heart damage in various ways. Oxidative stress, cell apoptosis, and fibrosis are closely correlated with I/R injury, which may further lead to heart failure 19. Apoptosis is the most important factor related to the loss of cardiomyocytes during I/R. The level of PUMA expression was very low under normal circumstances, while the expression of PUMA in I/R-treated heart tissues was upregulated 2. The I/R environment after heart transplantation induces elevated levels of PUMA expression in heart transplant tissues 20. PUMA has been shown to regulate apoptosis-promoting activity 21, 22. Therefore, it is reasonable to hypothesize that PUMA is a new therapeutic target for the regulation of apoptosis during cardiac I/R. In addition, ETS-1 upregulation mediates angiotensin II-induced cardiac fibrosis 6. ETS-1 participates in this process by regulating the expression of fibrotic matrix genes, such as collagen 1, collagen 3, and fibronectin 23. Consistent with previous observations, we demonstrated that I/R induced increases in oxidative stress, myocardial apoptosis and fibrosis, as shown by DHE staining, TUNEL assay and Masson's trichrome staining, respectively. In addition, we also showed that I/R-treated cardiac tissues and H/R-treated cardiomyocytes exhibited increased expression levels of PUMA and ETS-1. Importantly, we demonstrated that the downregulation of PUMA and ETS-1 expression by the transfection of siPUMA and siETS-1 directly prevented H/R-induced apoptosis and fibrosis. Moreover, conditioned medium from ADSCs decreased the expression of apoptosis- and fibrosis-related proteins. In this study, we demonstrated that targeting PUMA and ETS-1 has a cardioprotective effect against myocardial I/R injury by inhibiting apoptosis and fibrosis.

In recent years, MSCs have become a good source of cell therapy because of their easy availability 8. Compared with other types of MSCs, ADSCs have advantages in the treatment of myocardial infarction, liver fibrosis and oxidative damage 10. However, low survival rates, poor differentiation efficacy, and the potential carcinogenicity of implanted cells may impair the safety of stem cell-based therapies 24. Conditioned medium from ADSCs can be optimized by using increased concentrations and/or increasing the injection frequency, which is another method of directly transplanting stem cells 10. It is expected that the paracrine products secreted from stem cells will be converted into improved therapeutic agents 25, 26. The therapeutic effect of ADSC transplantation is mainly induced by paracrine-mediated cardioprotection and angiogenesis, while the role of ADSC differentiation and participation in angiogenesis is small 10. CM protects prolonged storage grafts from I/R injury 27. Human ADSC-CM has a therapeutic effect on mouse ischemic stroke models mainly by reducing nerve cell apoptosis and increasing endothelial cell proliferation 24. Bone marrow mesenchymal stem cell conditioned medium (BMSC-CM) reduced hypoxia-induced cardiomyocyte apoptosis 28. Our previous study showed that ADSC-CM protected against I/R-induced injury in skin flaps 29. Several studies have also shown that the injection of microparticles packaged from CM/secretome and coated with cell membranes of mesenchymal stem cells enhance the therapeutic benefits of CM after MI 30-32. In addition, the previous study reported that cardiac stem cell-derived exosomes conjugated with cardiac homing peptide alleviated I/R-induced cardiac injury 33. Another study showed that platelet-targeted delivery of peripheral blood mononuclear cells to the ischemic heart restores cardiac function after I/R injury 34. The present study showed that ADSC-CM treatment reduced the expression of apoptosis-related proteins, such as PUMA, p-p53, and BCL2, and decreased apoptosis. I/R increased the accumulation of excessive extracellular matrix, which leads to ventricular remodeling and irreversible myocardial fibrosis 35. In this study, ADSC-CM treatment significantly decreased the expression of ETS-1, fibronectin, and collagen 3, and attenuated cell fibrosis. Therefore, we demonstrated that ADSC-CM administration can play a beneficial role in I/R injury by reducing cardiac apoptosis and fibrosis.

The paracrine products produced by ADSCs include cytokines, growth factors, and miRNAs 11. In addition, exosomes secreted by most cell types, including mesenchymal stem cells, have beneficial effects on acute ischemia/reperfusion injury and can reduce the adverse remodeling in pigs after chronic MI 36, 37. A recent study showed that miR-21-5p dysregulation occurs in exosomes derived from heart failure patients, which can inhibit angiogenesis and cardiomyocyte survival 38. To explore the mechanism by which ADSC-CM protects cardiomyocytes from I/R damage, we studied this miRNA-mediated process. MiRNAs are usually released to recipient cells as key regulators 39. ADSC-CM reduced cardiomyocyte apoptosis, which may be related to the transfer of miRNAs from the conditioned medium to cardiomyocytes 10. In this study, we focused on miR-221/222, which is enriched in ADSC-CM, and found that the expression of miR-221/222 in ADSC-CM was closely related to the protective effect of ADSC-CM both *in vitro* and *in vivo*. MiR-221/222 suppresses apoptosis by targeting growth arrest-specific transcript 5 in breast cancer 40. PUMA has been reported to be the target of miR-221/222 in human epithelial cancers, human glioma cells, and multiple myeloma cells 14, 41, 42. In addition, miR-221/222 targets ETS-1 in endothelial cells to regulate angiotensin II-induced endothelial inflammation and migration 43. ETS-1 also plays a role in heart remodeling 6. MiR-221/222 is involved in ETS-1-regulated fibrosis 44. Here, we provide the first evidence that the expression levels of miR-221/222 in I/R-treated hearts and H/R-treated cardiomyocytes were decreased, while ADSC-CM increased these levels. The transfection of miR-221/222 mimics decreased I/R-induced cardiac injury. Using luciferase reporter assays, we proved that miR-221/222 of ADSC-CM could directly bind to the 3'-UTRs of PUMA and ETS-1 and inhibit the expression of PUMA and ETS-1. Moreover, compared with ADSC-CM, anti-miR-221/222 treatment had the opposite effects on PUMA and ETS-1 expression. Therefore, miR-221/222 is thought to inhibit PUMA and ETS-1 though sequence-specific target recognition and subsequently inhibit cardiomyocyte apoptosis and fibrosis. The present study provides evidence that PUMA and ETS-1 are direct targets of miR-221/222, and they act as apoptosis and fibrosis regulators in cardiomyocytes, respectively. MiR-221/222 in ADSC-CM exerts protective effects on I/R-treated hearts and H/R-treated cardiomyocytes both *in vitro* and *in vivo*.

p38 MAPK and NF-κB are frequently activated after cells are exposed to environmental stress, such as I/R-induced cardiac injury 45, 46. P38/NF-κB has been reported to be a mediator of stress-induced cell death and fibrosis 47, 48. P-p38/NF-κB participates in inflammation and apoptosis by regulating the expression of many downstream target genes, such as the apoptotic protein PUMA 2, 49, 50. The activation of NF-κB signaling leads to the upregulation of PUMA during I/R injury in heart transplantation 20. Inhibitors of p38/NF-κB expression appear to be downstream of PUMA inhibition 51, 52. Inhibition of p53-PUMA feedback loop activation prevents neuronal apoptosis and inflammatory responses by downregulating the NF-κB pathway in spinal cord I/R injury 52. In addition, the downstream signaling cascade of p38 MAPK and NF-κB induces ETS-1 expression 53, 54. Epidermal growth factor-induced ETS-1 regulates the expression of collagen I and III through the p38-MAPK signaling pathway in smooth muscle cells 55. Furthermore, the activation of NF-κB activates ETS-1 expression 56. Our previous study showed that activation of p38/NF-κB affects the expression of miR-221/222, thereby increasing ICAM-1 expression and monocyte adhesion in TNF-α-treated endothelial cells 57. The present study showed that the phosphorylation levels of p38 MAPK and NF-κB p65 in H/R-treated cardiomyocytes and I/R-treated mice were significantly increased compared to those of normal control mice, but ADSC-CM treatment significantly abrogated the increased phosphorylation levels of p38 MAPK/NFκB p65. We also demonstrated that miR-221/222 mimics inhibited I/R-induced p38/NFκB activation. Treatment with SB203580 (a p38 MAPK inhibitor) and Bay 11-7082 (a p65 inhibitor) markedly attenuated the expression of PUMA and ETS-1 in H9c2 cells exposed to the H/R environment. Our work demonstrated a new pathway by which miR-221/222 regulates cardiac apoptosis and fibrosis, namely, the p-p38/NF-κB pathway. We concluded that the protective effects of ADSC-CM rely on the miR-221/222/PUMA/ETS-1 pathway and p38 MAPK/NFκB signaling pathway.

In summary, this study provides the first evidence from *in vitro* and *in vivo* experiments confirming that the cardioprotective effect of ADSC-CM is mediated by increasing miR-221/222 expression and blocking p38/NF-κB phosphorylation, thus leading to decreased PUMA and ETS-1 expression in the context of ischemic injury. Exploring the active components and functional mechanisms of ADSC-CM is critical for the development of a new type of myocardial ischemia biotherapy. This study clarified the mechanism by which ADSC-CM protects the ischemic myocardium and provided a new method to reduce myocardial ischemic injury.
